# Supplementary material for: MiR-29b-1-5p is altered in BRCA1 mutant tumours and is a biomarker in basal-like breast cancer
Source: Oncotarget. 2018 Sep 11;9(71):33577–88. doi: 10.18632/oncotarget.26094 (PMC6173367; doi:10.18632/oncotarget.26094)
Supplement: Supplementary file 1 [file oncotarget-09-33577-s001.pdf]

MiR-29b-1-5p is altered in BRCA1 mutant tumours and is a biomarker in basal-like breast cancer

SUPPLEMENTARY MATERIALS

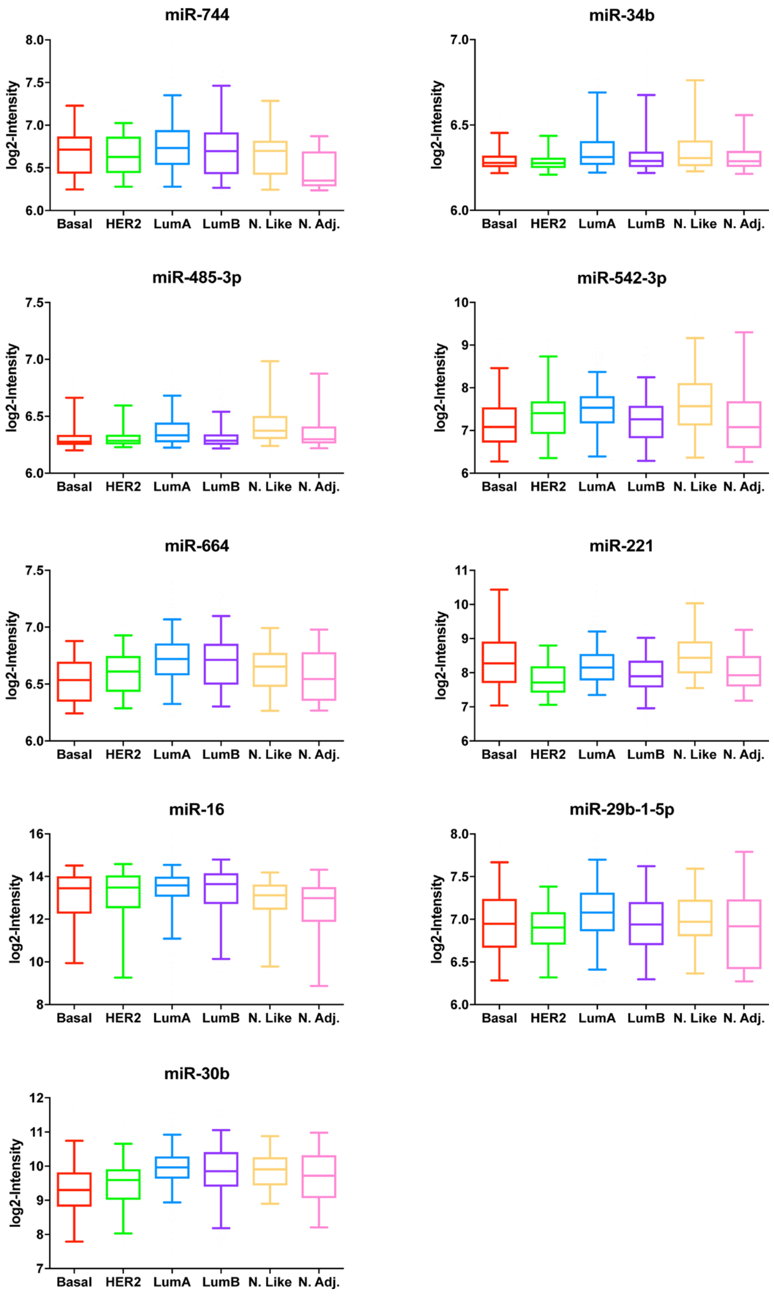

Supplementary Figure 1: Deregulated miRNAs are differentially expressed in breast cancer. RNA expression for the deregulated miRNAs across the PAM50 molecular subtypes of breast cancer. Expression are log2 normalised microarray intensities. Basal (basal-like, *n* = 179), HER2 (HER2-enriched, *n* = 112), LumA (Luminal A, *n* = 568), LumB (Luminal B, *n* = 354), N. Like (Normal-like tumours, *n* = 82) and N. Adj. (Normal-adjacent, *n* = 116). Box and whisker plots with 5–95 percentile are graphed.

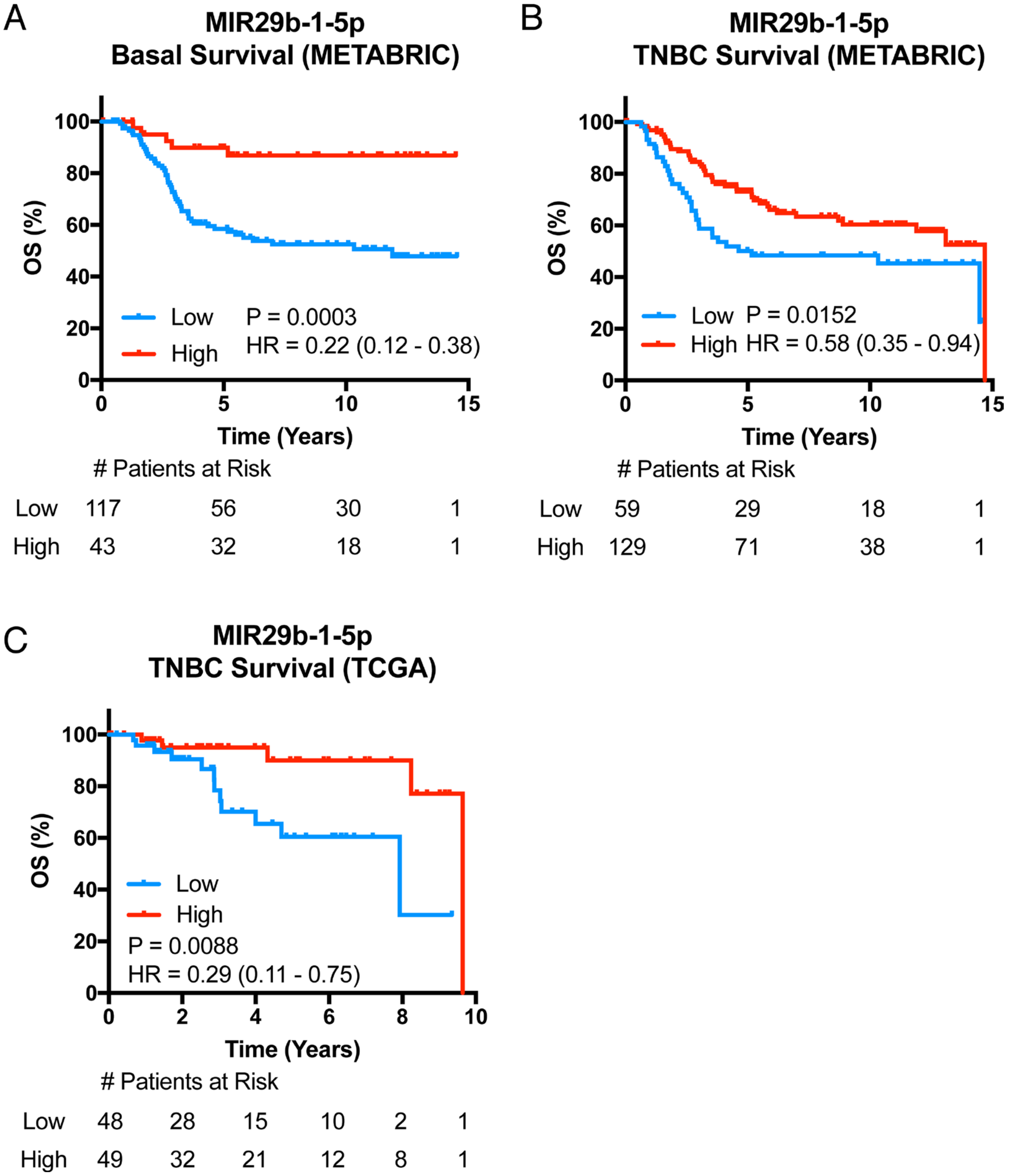

Supplementary Figure 2: MiR-29b-1-5p stratifies overall survival of basal-like and TNBC breast tumours. (**A**) Stratification of overall-survival for basal-like tumours based on expression of miR-29b-1-5p, High (43 patients) versus Low (117 patients). Data was sourced from METABRIC [43, 48]. (**B** and **C**) Stratification of overall-survival for triple-negative breast cancers (TNBC) in the METABRIC (A) and TCGA (B) cohorts, based on expression of miR-29b-1-5p, High (129 and 49 patients respectively) versus Low (59 and 48 patients respectively). Data sourced from KM Plotter [50]. Overall Survival (OS), Hazards Ratio (HR), Confidence Interval (CI).

Supplementary Table 1: Differentially expressed microRNAs from Brca1 knockout lactation murine mammary glands. See Supplementary_Table_1

Supplementary Table 2: *P*-values corresponding for Figure 1B

miRNA	*P*-value
miR-34b-5p	0.0004
miR-744-5p	0.0031
miR-485-3p	0.0003
miR-542-3p	0.0013
miR-664-3p	0.0004
miR-221-3p	0.0007
miR-16-5p	0.0126
miR-29b-1-5p	<0.0001
miR-30b-5p	0.0085

Supplementary Table 3: Predicted targets of human miR-29b-1-5p. See Supplementary_Table_3

Supplementary Table 4: miScript sequences for miRNA primers used for qRT-PCR

miRNA	Target sequence	Cat. No.
mmu-miR-206-3p	MIMAT0000239: 5'UGGAAUGUAAGGAAGUGUGUGG	MS00001869
hsa-miR-30b-5p	MIMAT0000420: 5'UGUAAACAUCCUACACUCAGCU	MS00003276
hsa-miR-744-5p	MIMAT0004945: 5'UGCGGGGCUAGGGCUAACAGCA	MS00010549
hsa-miR-664a-3p	MIMAT0005949: 5'UAUUCAUUUAUCCCCAGCCUACA	MS00014819
hsa-miR-16-5p	MIMAT0000069: 5'UAGCAGCACGUAAAUAUUGGCG	MS00031493
hsa-miR-485-3p	MIMAT0002176: 5'GUCAUACACGGCUCUCCUCUCU	MS00031885
hsa-miR-34b-5p	MIMAT0000685: 5'UAGGCAGUGUCAUUAGCUGAUUG	MS00031780
hsa-miR-542-3p	MIMAT0003389: 5'UGUGACAGAUUGAUAACUGAAA	MS00010073
hsa-miR-29b-1-5p	MIMAT0004514: 5'GCUGGUUUCAUAUGGUGGUUUAGA	MS00009289
hsa-miR-221-3p	MIMAT0000278: 5'AGCUACAUUGUCUGCUGGGUUUC	MS00003857
hsa-miR-29b-3p	MIMAT0000100: 5'UAGCACCAUUUGAAAUCAGUGUU	MS00006566
has_RNU6b-13		MS00014000
